# Mechanisms of neuroimmune gene induction in alcoholism

**DOI:** 10.1007/s00213-015-3906-1

**Published:** 2015-03-20

**Authors:** Fulton T. Crews, Ryan P. Vetreno

**Affiliations:** Bowles Center for Alcohol Studies, University of North Carolina at Chapel Hill, School of Medicine, CB# 7178, 1021 Thurston-Bowles Building, Chapel Hill, NC 27599-7178 USA

**Keywords:** TLR4, HMGB1, Ethanol, Cytokines, Microglia, RAGE, Gut permeability, Amphoterin, Innate immune, Alcohol use disorder, Frontal cortex

## Abstract

**Rationale:**

Alcoholism is a primary, chronic relapsing disease of brain reward, motivation, memory, and related circuitry. It is characterized by an individual’s continued drinking despite negative consequences related to alcohol use, which is exemplified by alcohol use leading to clinically significant impairment or distress. Chronic alcohol consumption increases the expression of innate immune signaling molecules (ISMs) in the brain that alter cognitive processes and promote alcohol drinking.

**Objectives:**

Unraveling the mechanisms of alcohol-induced neuroimmune gene induction is complicated by positive loops of multiple cytokines and other signaling molecules that converge on nuclear factor kappa-light-chain-enhancer of activated B cells and activator protein-1 leading to induction of additional neuroimmune signaling molecules that amplify and expand the expression of ISMs.

**Results:**

Studies from our laboratory employing reverse transcription polymerase chain reaction (RT-PCR) to assess mRNA, immunohistochemistry and Western blot analysis to assess protein expression, and others suggest that ethanol increases brain neuroimmune gene and protein expression through two distinct mechanisms involving (1) systemic induction of innate immune molecules that are transported from blood to the brain and (2) the direct release of high-mobility group box 1 (HMGB1) from neurons in the brain. Released HMGB1 signals through multiple receptors, particularly Toll-like receptor (TLR) 4, that potentiate cytokine receptor responses leading to a hyperexcitable state that disrupts neuronal networks and increases excitotoxic neuronal death. Innate immune gene activation in brain is persistent, consistent with the chronic relapsing disease that is alcoholism. Expression of HMGB1, TLRs, and other ISMs is increased several-fold in the human orbital frontal cortex, and expression of these molecules is highly correlated with each other as well as lifetime alcohol consumption and age of drinking onset.

**Conclusions:**

The persistent and cumulative nature of alcohol on HMGB1 and TLR gene induction support their involvement in alcohol-induced long-term changes in brain function and neurodegeneration.

## Introduction: microglia and innate immune genes

Microglia are tissue-specific monocyte-like cells of mesodermal origin (Ginhoux et al. 2010), whereas all other brain cells are derived from the neuroectoderm. Monocytes and tissue-specific monocyte-like cells (e.g., microglia) express innate immune signaling molecules originally characterized within the peripheral immune system. In the brain, microglia constitutively express Toll-like receptor (TLR) 4 and other innate immune receptors that are responsive to proinflammatory signals like high-mobility group box 1 (HMGB1) but are also responsive to neurotransmitters (Kettenmann et al. 2013).

Innate immune gene upregulation with rapid monocyte responses to infection was first characterized in blood as acute phase response proteins that today are known to include multiple cytokines, chemokines, proteases, cellular oxidases, and cytokine receptors. Acute phase responses and monocyte activation involve amplification in the expression of a large number of genes through kinase signaling pathways that converge on two distinct transcription factors: nuclear factor kappa-light-chain-enhancer of activated B cells (NF-κB) and activator protein-1 (AP-1). Both NF-κB and AP-1 induction promote the expression of innate immune cytokines (Li and Verma 2002; Valles et al. 2004), including tumor necrosis factor-alpha (TNFα) and interleukin-1beta (IL-1β) as well as upregulation of TLRs and other cytokine receptors. In addition, innate immune proteases and oxidases are induced, particularly cyclooxygenase (COX-2) and nicotinamide adenine dinucleotide phosphate oxidase (NADPH oxidase) as well as major histocompatibility (MHC) signaling molecules, such as beta-2 microglobulin. These monocyte-microglial-expressed proteins and their receptors are innate immune signaling molecules (ISMs) that are expressed in the brain (Blanco and Guerri 2007; Guerri and Pascual 2010; Valles et al. 2004). This review will refer to these brain signaling molecules as “neuroimmune” due to their characterization in the immune system of the brain, while recognizing that signaling across multiple unique brain cells differs from immune inflammation in response to infection.

**Fig. 1:**
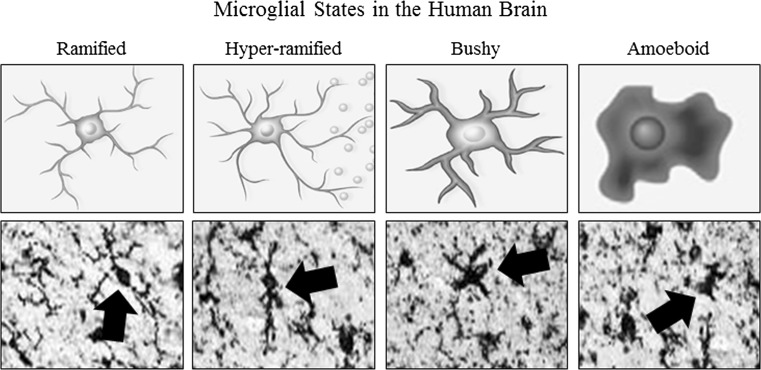
Activated morphology of microglia. Representative schematics and photomicrographs of human brain microglia (Iba1 immunohistochemistry) depicting morphological stages of microglial activation. Ramified or “resting” microglia are characterized by long, ramified processes with comparatively small cell bodies. Mildly activated hyper-ramified microglia are characterized by increased branching of processes as well as lengthening of processes and the secretion of proinflammatory cytokines (Beynon and Walker 2012). Bushy morphology is intermediate activation and is characterized by swollen, truncated processes, and enlarged cell bodies. Amoeboid or “phagocytic” microglia are characterized rounded macrophage-like morphology with no or few processes and are associated with maximal proinflammatory activation, oxidative-free radicals, and microglial apoptosis (Kreutzberg 1996; Raivich et al. 1999). Post-mortem human brains from controls and alcoholics contain all of these subtypes likely due in part to age-related changes in all individuals. Alcoholics have more microglial markers indicative of hyper-ramified morphology in alcoholics increasing staining compared to control ramified less dense staining. Figure adapted from He and Crews (2008) and Beynon and Walker (2012)

Brain neuroimmune signaling primarily involves monocyte-microglial innate immune signals and not adaptive immune antibodies. Although microglia are unique tissue-specific brain monocyte-like cells, similar to all monocytes, microglia undergo morphological changes that characterize stages of activation (Graeber 2010) (Fig. 1). Resting ramified microglia likely contribute trophic and other signals similar to the wound healing monocyte phenotype termed M2 that upon activation can become hyper-ramified, with secretion of proinflammatory cytokine signals (Beynon and Walker 2012). However, activated microglia do not necessarily always adopt an M1 phenotype as Marshall et al. (2013) found that young adult rats subjected to a 4-day binge model of alcohol led to partial microglial activation as evidenced by increased expression of OX-42 but not a fully activated phenotype characterized by expression of OX-6 or ED-1. This partial microglial action was accompanied by an increase in the anti-inflammatory cytokine IL-10 and no increase in proinflammatory cytokines IL-6 or TNFα. Further proinflammatory activation, known as M1 monocyte phenotype, involves expansion of processes to a “bushy morphology” and finally a “phagocytic” rounded morphology (Colton 2009). The relationship between morphological changes in monocyte-like cells including microglia and the secretion of ISM is poorly understood, although increased severity of pathology is associated with greater ISM induction and activated morphology.

**Fig. 2:**
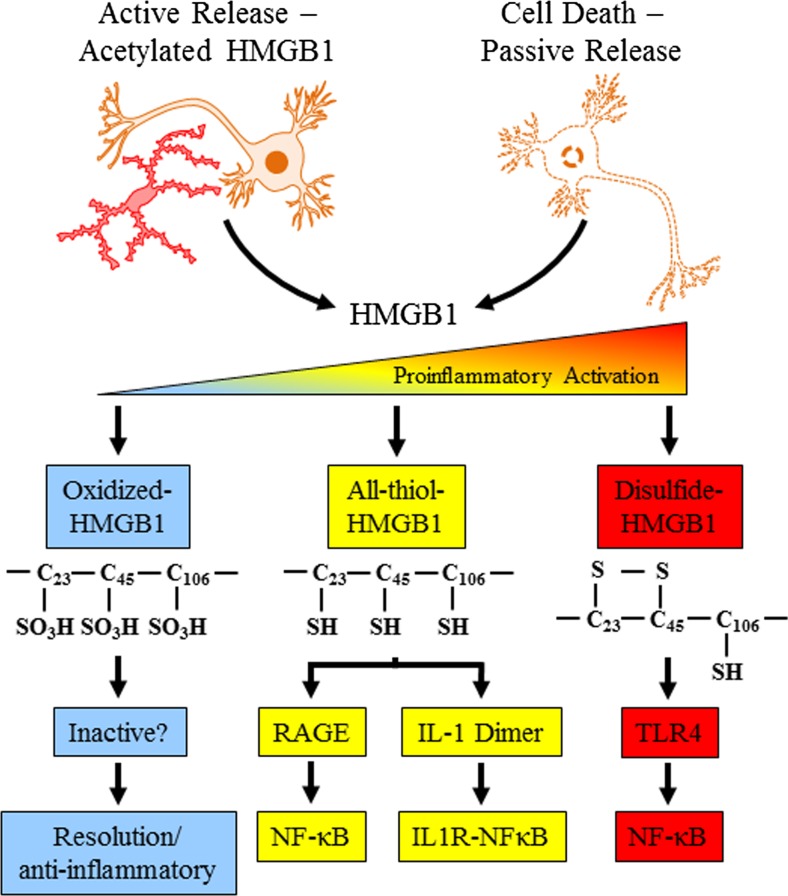
High-mobility group box 1 (*HMGB1*) is actively and/or passively released leading to multiple signaling pathways. Actively released HMGB1 from brain slice cultures found histochemical evidence of release from neurons by ethanol (Zou and Crews 2014), although HMGB1 release likely occurs from most brain cell types. Neurons and glia release HMGB1 during glutamate excitation (Maroso et al. 2010, 2011). HMGB1 is also released during necrotic cell death activating innate immune signaling. HMGB1 has multiple signaling mechanisms regulated by oxidation of cysteines. Fully oxidized HMGB1 (*blue*, *left*) does not activate proinflammatory signaling, although it may contribute to resolution of the proinflammatory state. HMGB1 in the all-thiol form (*yellow*, *middle*) is an agonist at the receptor for advanced glycation end-products (*RAGE*; Allette et al. 2014). All-thiol-HMGB1 also forms heterodimers with proinflammatory molecules such as interleukin-1beta (*IL-1β*; Wahamaa et al. 2011) with the HMGB1-IL1 heterodimer synergistically enhancing stimulation of the IL-1β receptor leading to proinflammatory gene induction through activation of NF-κB transcription (Venereau et al. 2012). Disulfide-HMGB1 (*red*, *right*) activates Toll-like receptor 4 (*TLR4*) also leading to nuclear translocation of nuclear factor kappa-light-chain-enhancer of activated B cells (*NF-κB*) and induction of proinflammatory cytokines. Adapted from Tang et al. (2012)

Like all monocytes, microglial activation can lead to NF-κB transcription of proinflammatory genes, which signal to other microglia as well as astroglia, oligodendroglia, and neurons, amplifying neuroimmune gene induction within and across cells by induction of TLRs and cytokine receptors, many belonging to the IL-1β receptor family that activate kinase cascades that converge on NF-κB (Blanco and Guerri 2007; Blanco et al. 2004, 2005; Fernandez-Lizarbe et al. 2009, 2013; Pascual et al. 2011b; Valles et al. 2004). The amplification of ISMs across cells and tissue can lead to pathology, and understanding the processes of monocyte signaling provides insight into microglial signaling in brain. The most severe acute example of monocyte activation during infection is sepsis. Sepsis and the systemic inflammatory response syndrome refer to a “cytokine storm,” which involves a pronounced increase in multiple proinflammatory cytokines and other ISMs that cause a potentially fatal innate immune reaction consisting of positive feed-forward loops between cytokines and immune and tissue cells that result in highly elevated cytokine blood levels, multi-organ failure, and death (Osterholm 2005). Models of sepsis that involve activation of an acute phase-like response lead to increased expression of multiple cytokines that are induced in distinct phases. During the initial phase, TNFα and IL-1β expression is increased during the first several hours after innate immune activation and then subside. The second phase involves HMGB1, which is an agonist at multiple receptors that contribute to further activation of proinflammatory cascades (Fig. 2). Disulfide-HMGB1 is a TLR4 agonist (Tang et al. 2012), and thiol-HMGB1 is an agonist at the receptor for advanced glycation end products (RAGE; Allette et al. 2014) and also dimerizes with proinflammatory molecules (Tang et al. 2012; Venereau et al. 2012), such as IL-1β (Wahamaa et al. 2011) that enhances IL-1β receptor induction of proinflammatory molecules through NF-κB. HMGB1 increases in blood approximately 16 h after infection in models of sepsis and persists for several days during which mice die. Mortality is prevented by anti-HMGB1 antibody treatment (Wang et al. 2001) consistent with HMGB1 induction of a “cytokine storm” by acting through multiple receptors that converge on proinflammatory NF-κB signaling. Survivors of sepsis models show prolonged increases in serum HMGB1 and cognitive deficits that are blunted with HMGB1 antibody treatment (Chavan et al. 2012). To model alcoholic hepatitis and alcohol-induced release of gut endotoxin, we systemically administer lipopolysaccharide (LPS) and polyinosinic:polycytudylic acid (poly I:C). Administration of these endotoxins systemically after ethanol treatment exacerbates the innate immune response. Acute binge drinking also increases serum endotoxin levels albeit at a lower level observed under septic conditions. High binge drinking doses cause the gut to become permeable or “leaky” (Ferrier et al. 2006). Only high doses of ethanol, e.g., at least 2–3 g/kg ETOH intragastric doses (Ferrier et al. 2006), potentiate gut innate immune signaling, disrupting gut tight junctions, and opening sites that allow the gut biome bacteria and their endotoxins to enter the portal circulation leading to the liver where they can initiate a proinflammatory response (Sims et al. 2010). Released LPS potentiates alcohol-induced liver inflammation and secretion of proinflammatory cytokines, including the proinflammatory cytokine TNFα, which is released into the blood. Proinflammatory cytokines in the blood are transported across the blood–brain barrier (Banks and Erickson 2010; Qin et al. 2007) such that both cytokines and alcohol enter the brain where they induce neuroimmune activation.

**Fig. 3:**
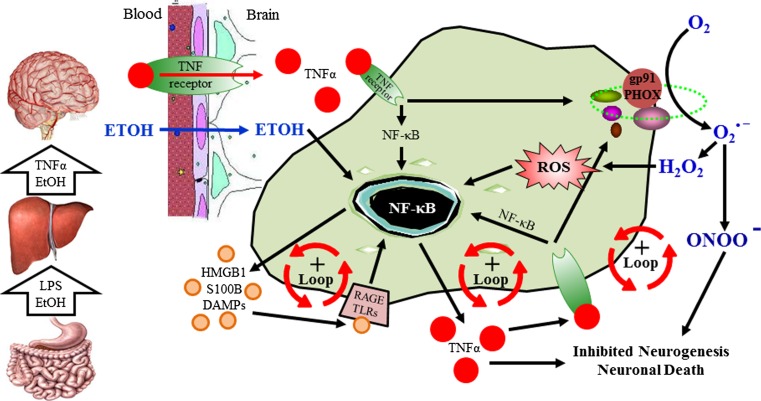
Ethanol in the gut causes leakage of bacterial products into the portal vein increasing hepatic TNFα release into the blood which induces neuroimmune gene expression in the brain. High doses of consumed alcohol in the gut (i.e., at least 2–3 g/kg ETOH intragastric doses [Ferrier et al. 2006]) increases permeability allowing bacterial products such as endotoxin-lipopolysaccharide (*LPS*) to enter portal circulation. Alcohol and LPS enter portal circulation leading to induction of liver tumor necrosis factor-alpha (*TNFα*) and other proinflammatory cytokines that are released into the blood and enter the brain through cytokine-specific receptor transport (e.g., the TNFα receptor) (See Qin et al. 2007 for details). This activates positive loops of proinflammatory gene induction in the brain that all converge upon nuclear translocation of nuclear factor kappa-light-chain-enhancer of activated B cells (*NF-κB*) that amplify through autocrine and paracrine positive loops. Further, ethanol causes nuclear release of high-mobility group box 1 (*HMGB1*) and other danger associated molecular patterns (*DAMPs*) that enters the extracellular space activating Toll-like receptors (*TLRs*) and the receptor for advanced glycation end products (*RAGE*) in an autocrine and paracrine fashion further amplifying NF-κB transcription. In parallel, proinflammatory oxidases form reactive oxygen species (*ROS*) also amplify activation of NF-κB transcription that can contribute to inhibition of neurogenesis and cause neuronal death

Innate immune signaling molecules in the brain appear to contribute to both brain health and pathology. Indeed, recent studies find that MHC molecules contribute not only to most neurodegenerative diseases (Gage 2002; Glass et al. 2010) as well as alcohol and drug dependence (Crews 2012) but are also critically involved in brain development (Huh et al. 2000). Within the brain microglia, innate immune cytokines, such as TNFα, IL-1β, and HMGB1 as well as TLRs, purinergic receptors (e.g., P2X7), various cytokine receptors, and innate immune proteases and oxidases, all amplify through NF-κB and AP-1 loops that confound studies that are focused on studying a single neuroimmune signaling molecule (Fig. 3). NF-κB regulates the transcription of proinflammatory innate immune genes as well as many other genes (Perkins 2007). Ethanol increases NF-κB–DNA binding and expression of multiple innate immune genes including proinflammatory cytokines, TNFα, IL-1β, and MCP-1, the proinflammatory oxidase, iNOS, and proteases TACE and tPA (Zou and Crews 2010). Previously, we found that ethanol increased NF-κB p65 nuclear immunohistochemistry consistent with NF-κB p50⁄p65 subunit nuclear translocation and transcription activation. Similarly, we found that antibodies to p50 or p65 super-shifted EMSA gels, suggesting that ethanol increased brain NF-κB p65–p50 heterodimer–DNA binding (Zou and Crews 2006). Taken together, ethanol-induced NF-κB–DNA binding and target gene expression support ethanol activation of NF-κB transcription of proinflammatory genes. However, using an ELISA-based DNA binding analysis, we found large increases in NF-κB subunit p50 protein but not NF-κB p65 protein. A similar finding has been reported for prefrontal cortex gene expression in the post-mortem human alcoholic brain (Okvist et al. 2007). Array analysis of gene expression in post-mortem alcoholic prefrontal cortex found 479 transcripts with NF-κB–DNA binding sites that were generally upregulated, with analysis of NF-κB subunit proteins indicating NF-κB p50 was the dominant subunit expressed in human alcoholic brain (Okvist et al. 2007). Although homodimers of NF-κB p50 inhibit transcription (Perkins 2007), increases in NF-κB p50 protein are often associated with increased transcription. Several mechanisms are involved in increased NF-κB p50 activation of transcription including protease processing of inhibitory NF-κB p105 to transcriptionally active NF-κB p50 (Hoffmann et al. 2006) or through NF-κB p50 homodimer association with BCL3, atypical IκB, and other proteins that activate gene transcription involving NF-κB p50 (Ghosh and Hayden 2008). Thus, an increase in NF-κB p50 is consistent with increased NF-κB gene transcription. Although transcription is complex, ethanol-induced progressive increases in NF-κB–DNA binding and increased transcription likely represent one mechanism of ethanol induction amplification of brain innate immune genes. The mechanisms that regulate innate immune gene induction in various brain cells that contribute in vivo to amplification of specific innate immune genes are poorly understood. In the current review, literature pertaining to alcoholism and innate immunity was reviewed using the PubMed search engine, and this review will focus on two mechanisms of ethanol sensitization of microglia and induction of neuroimmune genes. The first involves a systemic mechanism whereby blood innate immune signaling molecules induce brain neuroimmune genes and a second local mechanism involving neuronal–glial signaling through neuroimmune genes that regulate neuronal excitability. It should be noted that neuroimmune activation appears to be involved in the later stages of heavy drinking and that binge drinking is required to activate the innate immune system.

## Ethanol-induced blood innate immune signals activate brain neuroimmune signaling

Acute binge drinking increases blood cytokines in normal healthy humans (Bala et al. 2014). Recent studies indicate that ethanol in the gut releases HMGB1 (Ge et al. 2014) which activates TLR4 causing the gut to leak endotoxin LPS-like bacterial products that stimulate proinflammatory cytokine induction in the liver (Ge et al. 2014) thereby increasing circulating blood cytokines (see Gao et al. 2011 for a review) (Fig. 3). One mechanism involves ethanol causing the gut to become more permeable or “leaky.” Indeed, this occurs primarily with high doses of ethanol (i.e., at least 2–3 g/kg ETOH intragastric doses [Ferrier et al. 2006]) that activate gut innate immune signaling through disruption of gut tight junctions that allows the gut microbiome bacteria and their endotoxins to enter portal circulation leading to the liver inducing an innate immune response in blood (Sims et al. 2010). Alcoholism is associated with liver disease resulting in many alcoholics having elevated levels of blood cytokines. However, in healthy adults, an acute alcohol binge dose also increases blood cytokines (Bala et al. 2014), and in human alcoholics without liver disease, gut permeability is increased during active drinking with increased blood endotoxin and proinflammatory cytokines (Leclercq et al. 2012). Interestingly, a 3-week period of abstinence led to a resolution of gut permeability, but levels of circulating proinflammatory cytokines (i.e., TNFα and IL-6) remained elevated across abstinence. Furthermore, the authors found that serum levels of circulating cytokines were correlated with levels of depression and alcohol craving. These data suggest that ethanol-induced gut permeability results in a peripheral blood innate immune response that impacts brain and behavior (Leclercq et al. 2012). Systemic infection is associated with a constellation of sickness behaviors including cognitive and emotive dysfunction, depression, fever, lethargy, and impaired memory (Dantzer et al. 2008). In a randomized human study, subjects that received a typhoid vaccination had elevated levels of circulating IL-6 that was associated with sickness behavior (Harrison et al. 2009a, b). Interestingly, the IL-6-induced reductions of mood were associated with increased activity in the anterior cingulate cortex during emotional face processing as assessed using fMRI. Interestingly, connectivity of the anterior cingulate cortex, which has been implicated in the etiology of depression, with the amygdala, medial prefrontal cortex, and nucleus accumbens was reduced and associated with circulating IL-6 (Harrison et al. 2009a). Proinflammatory cytokines in the blood are actively transported across the blood–brain barrier (e.g., TNF receptor [Banks and Erickson 2010; Qin et al. 2007]) and also stimulate endothelial cells to release cytokines in brain (Watkins et al. 1995) (see Fig. 3). Thus, acute ethanol exposure can induce a blood cytokine response by increasing endotoxin leak from the gut that can lead to changes in the brain and behavior.

Induction of acute increases in blood levels of TNFα, other cytokines, and ISM can have long-lasting effects on the brain. For instance, Qin et al. (2007) found that a single systemic dose of LPS increased mRNA and protein levels of TNFα in parallel in the liver, blood, and brain, but the liver and blood response subsided after 12–24 h, whereas the brain response persisted for at least 10 months. However, in transgenic mice lacking blood–brain barrier TNF receptor transporters, a single systemic dose of LPS increased TNFα levels in the blood but not in the brain suggesting that TNFα transport by its receptor across the blood–brain barrier is necessary for activating proinflammatory neuroimmune responses in the brain. As mentioned above, proinflammatory gene expression in blood and brain parallels each other at early time points. Surprisingly, the increase in proinflammatory gene expression in the brain persisted for months leading to degeneration of dopaminergic neurons of the substantia nigra that could contribute to dopamine hypofunction leading to sensitization of the response to the rewarding effects of ethanol (Blednov et al. 2011; Qin et al. 2007). Although the binge ethanol-induced liver and blood responses are not as pronounced as LPS, chronic ethanol pretreatment sensitizes systemic and brain proinflammatory cytokine responses to a single systemic dose of LPS following the conclusion of ethanol treatment through TLR4 (Qin et al. 2008) and to a single systemic dose of poly I:C through TLR3 (Qin et al. 2013). Ethanol appears to sensitize microglia by upregulating multiple innate immune signaling molecules, including TLR receptors (Crews et al. 2013). Chronic ethanol treatment of mice for 10 consecutive days (5.0 g/kg, i.g.) increases brain expression of the proinflammatory cytokine monocyte chemotactic protein-1 (MCP-1) that persists for at least 1 week of abstinence following ethanol treatment (Qin et al. 2008). Exposure of C57BL/6J mice to 10 daily doses of ethanol followed by a single systemic dose of LPS resulted in a potentiation of proinflammatory cytokine induction in the liver, blood, and brain, resulting in persistently higher brain expression long into abstinence relative to LPS alone-treated animals (Qin et al. 2008). In these studies, ethanol sensitized mice to the LPS-induced neuroimmune response that resulted in sustained increases in multiple proinflammatory cytokines (e.g., TNFα, IL-1β, and MCP-1) in the brain but not in the liver. While the mechanism(s) of the sustained brain response and transient liver response are not clear, we found that IL-10, an anti-inflammatory factor that inhibits NF-κB, is increased in the liver 1 week after alcohol treatment but decreased in the brain (Qin et al. 2008). This finding is consistent with anti-inflammatory mechanisms contributing to the reversal of the liver response. Bacterial products enter portal circulation activating Kupffer cells (i.e., liver monocytes) that produce cytokines, including TNFα, that can be transported into the brain where they activate brain neuroimmune signaling that persists for long periods (Qin et al. 2007). Thus, the spread of a systemic innate immune response to brain represents a mechanism of brain neuroimmune gene induction.

## Microglia and alcohol in the brain

Ethanol-induced hyper-ramified microglia have been implicated in alcohol-induced activation of neuroimmune signaling pathways in the brain. In rats, intermittent and chronic ethanol exposure sensitize microglia, priming them for further activation and increasing expression of proinflammatory cytokines providing indirect evidence for the role of microglia in alcohol-induced neuroinflammation and neurotoxicity (Alfonso-Loeches and Guerri 2011; Zhao et al. 2013). Alcohol activates microglia via TLRs (Alfonso-Loeches and Guerri 2011). Specifically, TLR4 expression and TLR4/TLR2 association on cultured microglia appear to be necessary for alcohol-induced microglia activation with the production of inflammatory mediators and cortical neuronal apoptosis (Fernandez-Lizarbe et al. 2009, 2013). In an in vitro study (Boyadjieva and Sarkar 2013b), application of microglial-conditioned media enhanced ethanol-induced apoptosis of cultured hypothalamic neurons. Interestingly, neutralization of TNFα abolished the neuronal cell death induced by microglial-conditioned media, suggesting that microglial production of TNFα plays a key role in ethanol-induced neurotoxicity in developing neurons. Additionally, they found that ethanol induces oxidative stress in neurons by increasing the cellular production of O_2_^−^, reactive oxygen species (ROS), and nitrite while decreasing levels of the antioxidant glutathione (GSH) and the cellular activity of other antioxidative enzymes, including glutathione peroxidase, catalase, and superoxide dismutase. Furthermore, treatment with either a synthetic superoxide dismutase/catalase mimetic (*EUK*-*134*) or a water-soluble analog of vitamin E (Trolox), both of which are well-known antioxidants, protected developing hypothalamic neurons from oxidative stress and cellular apoptosis caused by ethanol-treated microglia media. These findings are consistent with proinflammatory loops of positive feed-forward amplification of neuroimmune signaling with multiple components, including TLR receptors and proinflammatory cytokines that contribute to neurodegeneration (Fig. 3). Further, the P2X7R, which is a member of the purinergic P2X family of ATP-gated ion channels, is highly expressed on microglia and activation of these receptors is associated with release of the proinflammatory cytokines IL-1β (Ferrari et al. 1997; Lister et al. 2007) and TNFα (Hide et al. 2000; Lister et al. 2007). The functional responses of P2X7R activation by ATP are associated with ongoing cellular damage and chronic brain inflammation. Indeed, recent experimental evidence indicates that stimulation of P2X7Rs mediate ATP-induced apoptosis through microglial production of superoxide (Parvathenani et al. 2003; Raouf et al. 2007). Interestingly, the P2X7 receptor might also play a role in microglial proliferation since TNFα application to hippocampal-entorhinal cortex slice culture led to an increased in proliferating microglia (Zou et al. 2012). Thus, there are numerous processes through which alcohol induces reactive oxygen species.

The systemic increases in proinflammatory signals broadly activate neuroimmune signals across the brain (Sugama et al. 2009). In addition to neuroimmune cytokines, oxidases such as COX-2, nitric oxide synthetasem and NADPH oxidase, which includes phargocytic oxidase (e.g., gp91^PHOX^, which was classically found increased in monocyte with phagocytic morphology) form ROS that can contribute to neurotoxicity (Takeuchi 2010). Reactive oxygen species are oxidative molecules that oxidize proteins, break down cell membranes, induce cell death, and activate NF-κB as a component of proinflammatory amplification within and across cells (Fig. 3). NADPH oxidase, a multi-subunit enzyme that catalytically makes superoxide, is increased in the frontal cortex by both a single systemic dose of LPS and 10 days of ethanol treatment (5.0 g/kg, i.g.), particularly gp91^PHOX^, which is the superoxide forming subunit (Qin and Crews 2012). These findings are consistent with oxidative stress, through innate immune gene induction, contributing significantly to alcoholic brain damage in the orbitofrontal cortex. Qin et al. (2013) found that a single systemic dose of LPS induces microglial activation, NADPH oxidase, and oxidative stress that persist for at least 20 months and leads to persistent neuroimmune gene induction and a progressive persistent neurodegeneration. The prolonged and persistent induction of NADPH oxidase and oxidative stress in the brain could contribute to the long-lasting increases in NF-κB transcription since oxidative free radicals can activate NF-κB.

In addition to generation by activated microglia, ROS are created as a natural by-product of alcohol metabolism and by increased cellular respiration thereby creating an environment of increased oxidative stress in the brain and glial cells that is conducive to neuronal cell death (Guerri et al. 1994; Montoliu et al. 1995). Several studies implicate microglia in alcohol-induced production of ROS and consequent neurotoxicity. For instance, Qin and Crews (2012) found that mice exposed to chronic 10-day ethanol treatment (5.0 g/kg, i.g.) evidence increased expression of NADPH oxidase (an enzyme that produces ROS), O_2_^−^, microglial activation, and cell death in cortical and hippocampal brain regions. Interestingly, inhibition of NADPH oxidase decreased O_2_^−^, microglial activation, and neuronal cell death, linking alcohol-induced microglial activation and neurotoxicity. Surprisingly, induced NADPH oxidase in the brain was found in neurons in both mice exposed to chronic 10-day ethanol treatment (5.0 g/kg, i.g.) and post-mortem human alcoholic orbitofrontal cortex (Qin and Crews 2012) consistent with neuroimmune signaling amplifying across brain cell types. In agreement with the in vivo findings, in vitro studies reveal that ethanol (25, 50, and 100 mM ethanol) dose-dependently increased induction of oxidative stress (i.e., O^2−^, ROS, and nitrite) and apoptotic cell death in neuronal hypothalamic primary cultures. Interestingly, ethanol-activated microglial-conditioned media potentiated ethanol-induced production of ROS and oxidative stress in cultured hypothalamic neuronal cells leading to increased apoptotic cell death (Boyadjieva and Sarkar 2013b). Therefore, ethanol-activated neuroimmune signaling may produce ROS and nitrite that decrease the cellular activity of anti-oxidative enzymes and increasing expression of neuroimmune molecules in neurons that contribute to neurodegeneration.

The mechanisms of alcohol-induced neurodegeneration are complex involving multiple neuroimmune signals as well as alterations in trophic signals (Crews and Nixon 2009; Guadagno et al. 2013). Ethanol induces IL-1β and IL-6 as well as transforming growth factor-β1 (TGF-β1; Alfonso-Loeches et al. 2010; Chen et al. 2006). One of the cellular signaling mechanisms by which alcohol induces neuronal apoptosis involves increased neuronal release of TGF-β1. It has been shown that alcohol-induced increases of TGF-β1 levels in neuronal cells is accompanied by increased expression of transcription factor E2F1 (overexpression sensitizes cells to apoptosis), reduced expression of cyclin D1 and cyclin-dependent kinase-4 (key regulator of cell cycle progression), elevated levels of mitochondrial pro-apoptotic proteins bak, bad, and bcl-xs, lowered levels of the anti-apoptotic protein bcl-2, increased production of apoptotic enzyme caspase-3, and increased neuronal cell death (Chen et al. 2006; Kuhn and Sarkar 2008). Hypothalamic neuronal cell cultures following treatment with ethanol-activated microglial-conditioned media showed decreased production levels of cyclic adenosine monophosphate (cAMP) and brain-derived neurotropic factor (BDNF; Boyadjieva and Sarkar 2013a). Further, treatment with BDNF or dibutyryl cAMP decreased ethanol-activated microglial-conditioned medium-induced changes in intracellular free radicals, ROS, and O_2_ as well as nitrite, GSH, and catalase. Therefore, ethanol by increasing the production of microglial-derived factors reduces cellular levels of cAMP and BDNF leading to an increase in cellular oxidative status and apoptosis of neuronal cells. Although ethanol-induced sensitization of microglia leads to an increase in the production of ISMs while also decreasing trophic support that clearly contributes to altered neuronal vitality and increase neuronal death, further studies are needed to identify all the mechanisms by which ethanol-activated signaling induces neurodegeneration.

## Ethanol and neuronal excitation release HMGB1 triggering neuroimmune activation

**Fig. 4:**
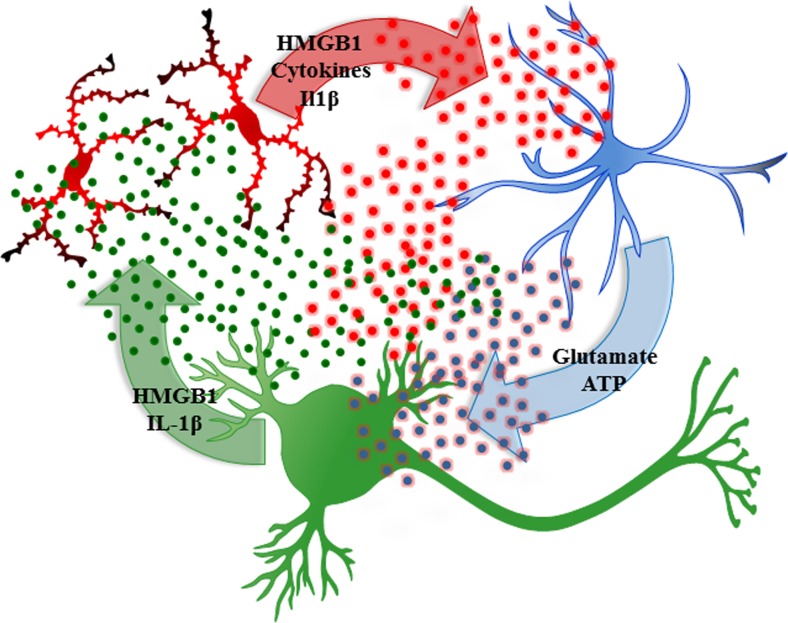
Neuronal excitability releases HMGB1, which increases cytokine secretion by microglia that activate astrocytes that increase glutamate increasing neuronal excitability through glutamate, HMGB1, and other signals. Glutamate, alcohol, and other factors release HMGB1 from neurons causing microglia to become hyper-ramified, resulting in their release of high-mobility group box 1 (*HMGB1*) and other proinflammatory cytokines. These innate immune signaling molecules stimulate astrocytes reducing glutamate uptake (Zou and Crews 2005) and increasing astrocyte release of glutamate that induce neuronal excitability causing further increases in HMGB1 release. Increased neuronal hyperexcitability can contribute to potentiation of specific neuronal connectivity and/or to excitotoxic neuronal cell death. Figure adapted from Kettenmann et al. (2013)

**Fig. 5:**
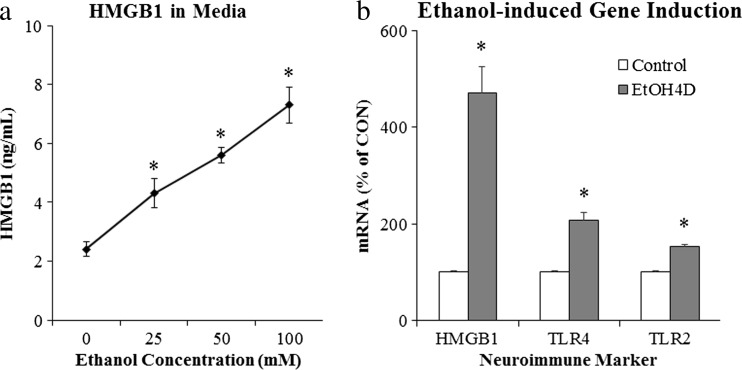
Ethanol releases high-mobility group box 1 (*HMGB1*) leading to activation of neuroimmune signaling. Ethanol causes the release of HMGB1 into the media from hippocampal-entorhinal cortex (*HEC*) slice culture. **a** Ethanol causes dose-dependent increase of HMGB1 release into culture media, relative to controls (* *p* < 0.05, *n* = 3). **b** Western blot analysis of the whole cell lysate revealed that HMGB1 protein content increased progressively over time in response to ethanol treatment (100 mM EtOH). **c** RT-PCR analysis found that ethanol treatment (100 mM) for 4 days significantly increased Toll-like receptor (*TLR*) 2, TLR4, and HMGB1 mRNA in HEC slice culture (* *p* < 0.05, *n* = 3). Data are adapted from Zou and Crews (2014)

A second mechanism of neuroimmune activation in brain involves neuronal activation of adjacent microglia and astrocytes (Crews and Vetreno 2014; Sugama et al. 2009). This form of neuroimmune activation in specific neuronal nuclei may represent a form of neuroplasticity. Experience-induced neuroimmune gene induction can lead to long-lasting changes in neuronal excitability that could contribute to neuroplasticity similar to synaptic long-term potentiation. Pathological increases in excitability could contribute to mental diseases as well as increasing sensitivity to excitotoxic neuronal death. Excited neurons release HMGB1, also known as amphoterin (Huttunen and Rauvala 2004), which stimulates microglia thereby increasing NF-κB transcription of proinflammatory cytokines (Crews et al. 2013; Zou and Crews 2014) as well as increasing neuronal excitability (Maroso et al. 2010). Microglia and astrocytes release cytokines increasing neuronal excitability in part due to reduced glial uptake of glutamate (Zou and Crews 2005) and increased glial release of glutamate (Fig. 4) as well as neuronal HMGB1-TLR4 signaling that enhances neuronal sensitivity to glutamate (Maroso et al. 2010). Ethanol-induced release of HMGB1 (Fig. 5) likely contributes to adaptive changes in the brain that contribute to alcoholic neuropathology, both the behavioral pathology as well as neurodegeneration. However, it is still a matter of debate whether neurons express the TLR4 receptor as our group and others report evidence of neuronal TLR4 (Okun et al. 2009; Vetreno et al. 2014; Vetreno and Crews 2012) using immunohistochemistry, but other studies employing fluorescence-activated cell sorting techniques suggest that they are mostly expressed by glial cells (Schwarz and Bilbo 2013). Ethanol-induced release of HMGB1 results in the induction of multiple neuroimmune molecules including TLR4 and TLR3 receptors as well as increased HMGB1 expression (Fig. 5). The induction of agonists and receptors is characteristic of innate immune responses that likely contribute to amplification and persistence of innate immune gene induction in the brain. Actively released HMGB1 is acetylated, and we discovered that ethanol increases HMGB1 acetylation through altered histone deacetylases. Acetyl-HMGB1 is found in cytosolic vesicles that is released during neuronal activation or treatment with ethanol (Zou and Crews 2014). Release of acetyl-HMGB1 by ethanol is consistent with active neuronal release since cell death released HMGB1 is not acetylated. The importance of ethanol-induced release of HMGB1 and activation of TLR4 became apparent in large part due to the elegant experiments of Consuelo Guerri’s laboratory. These studies found that ethanol treatment induces neuroimmune genes in microglia and astrocyte cultures as well as in vivo in mice but not in transgenic cells or mice that lack TLR4 receptors (Alfonso-Loeches et al. 2010; Blanco et al. 2005; Fernandez-Lizarbe et al. 2009; Pascual et al. 2011a; Valles et al. 2004). The TLR4 receptor is constitutively expressed on microglia, making them key components of drug-induced neuroimmune activation (Fernandez-Lizarbe et al. 2009; Schwarz and Bilbo 2013). More recent studies by Guerri’s laboratory found that TLR4 is also integral to ethanol-induced cortical neuronal death (Alfonso-Loeches et al. 2010), dopamine release (Pascual et al. 2009), damage to white matter (Alfonso-Loeches et al. 2012), and other pathologies associated with chronic alcohol-induced changes in the brain (Pascual et al. 2011a). In hippocampal-entorhinal cortex slice culture studies, ethanol treatment increases innate immune gene expression in a time-dependent fashion similar to responses to LPS or IL-1β administration, although ethanol induces a much smaller response in comparison (Crews et al. 2013; Zou and Crews 2012). Primary culture studies allow for cell type-specific analysis of the effects of ethanol on neuroimmune gene induction. However, these culture techniques are limited in that they do not allow for assessment of the interaction of neurons with glia. Indeed, by using hippocampal-entorhinal cortex slice culture, which include neurons as well as glial cells, we found that HMGB1 activity through TLR4 is critically involved in the modulation of the innate immune response to ethanol (Zou and Crews 2014). These studies support the hypothesis that HMGB1-TLR4 signaling underlies many of the effects of alcohol on the brain. Although the culture studies indicate that ethanol can induce neuroimmune genes in glial (Blanco et al. 2005; Fernandez-Lizarbe et al. 2009) and brain slice cultures (Zou and Crews 2010b, 2014), the in vivo studies in transgenic mice lacking TLR4 receptors (Alfonso-Loeches et al. 2010; Pascual et al. 2011a) likely blunt both ethanol-induced systemic blood and the local neuronal responses. These studies support HMGB1-TLR4 signaling in neuroimmune gene induction by neuronal excitation and ethanol. The loss of ethanol-induced dopamine responses in mice lacking TLR4 receptors is consistent with HMGB1-TLR4 induction of neuroimmune genes contributing to the development of alcoholism as well as alcoholic neurodegeneration (Crews et al. 2011).

## Ethanol induction of HMGB1-TLR signaling in the brain

Numerous studies support a significant role for neuroimmune genes in contributing to alcoholism (Blednov et al. 2012; Crews and Vetreno 2014; Crews et al. 2011; Osterndorff-Kahanek et al. 2013). Human genetic studies find that polymorphisms of genes encoding IL-1β and other neuroimmune genes are associated with susceptibility to the development of alcoholism (Crews 2012; Marcos et al. 2008; Pastor et al. 2005). Neuroimmune gene expression is increased in human alcoholic brains and correlates across rodent lines bred for high and low alcohol consumption as models of alcoholism (Flatscher-Bader et al. 2005; Liu et al. 2008; Mulligan et al. 2006). Further, LPS treatment increases brain immune gene expression, and LPS treatment of high alcohol drinking C57BL/6J mice results in a further increase in alcohol consumption and preference that lasts for months (Blednov et al. 2011). The mechanism of increased alcohol drinking could involve neuroimmune-induced dopamine hypofunction in the ventral tegmental area sensitizing the brain to ethanol reward (Blednov et al. 2011). Transgenic knock down of certain neuroimmune genes in mice (Blednov et al. 2005, 2012) and targeted disruption of TLR4 in the central amygdala (Liu et al. 2011) reduced alcohol consumption. In addition, pharmacological suppressions of various neuroimmune signaling pathways reduce alcohol intake in different animal models (Bell et al. 2013; Mayfield et al. 2013). These studies suggest that neuroimmune gene induction contributes to increased alcohol drinking and alcoholism.

Studies investigating the mechanism of ethanol induction of proinflammatory genes in the brain have led to the discovery that chronic ethanol increases expression of TLRs as well as the TLR4 receptor agonist HMGB1 (see Fig. 5). Ethanol treatment of mice modeling 10 days of binge drinking (Crews et al. 2013) and in vitro treatment of rat brain slice cultures with ethanol for 4 days (Zou and Crews 2014) leads to increased expression of HMGB1, TLR2, TLR3, and TLR4 mRNA, with immunohistochemistry suggesting that the induction occurs largely in neurons (Crews et al. 2013). Studies in post-mortem human alcoholic brain also find increased expression of HMGB1, TLR2, TLR3, and TLR4 (Crews et al. 2013). Increased expression of receptors and agonists are common in innate immune signaling and contribute to amplification within and across cells. Ethanol induction of HMGB1 and TLR likely contributes to amplification of neuroimmune gene induction in concert with neuronal excitability (Fig. 4). Ethanol treatment of brain slice cultures finds that HMGB1 antagonists, siRNA TLR4 knockdown, and antagonists block ethanol induction of proinflammatory genes (Crews et al. 2013; Zou and Crews 2014). These studies suggest that HMGB1-TLR signaling is central to ethanol induction of neuroimmune genes. However, TLRs and many cytokine receptors are within the IL1-receptor family and share kinase signaling cascades in the brain and glial cells that all converge upon NF-κB (Blanco et al. 2005, 2008) confounding which signals might be first. Interleukin-1β-IL1 receptor signaling is linked to HMGB1-TLR signaling (Maroso et al. 2011). Interleukin-1β induction involves formation of the inflammasome, unique intracellular multi-protein organelles involved in the synthesis and secretion of IL-1β. We found that ethanol treatment of hippocampal brain slice cultures increased expression of IL-1β and NLRP-inflammasome proteins as well as increased expression in post-mortem alcoholic human hippocampus that with HMGB1-TLR signaling contributed to ethanol-induced inhibition of neurogenesis (Zou and Crews 2012). Although the mechanisms underlying ethanol-induced innate immune gene induction in the brain are complex involving many neuroimmune genes, HMGB1, TLR, and other neuronal–glial neuroimmune signaling molecules contribute to the neurobiology of alcoholism (Vetreno and Crews 2014).

## Persistence of neuroimmune gene induction in the brain

Although innate immune signaling in monocytes has many similarities with neuroimmune signaling in microglia, as mentioned earlier, one difference appears to be that neuroimmune action persists for long periods once activated. LPS treatment of mice, which models the gut release of endotoxins (e.g., LPS) and human alcoholic hepatitis, results in rapid increases in proinflammatory cytokines in the liver, blood, and brain, with liver and blood levels returning within 24 h, whereas brain microglial activation persists for months that after 7 and 10 months results in a progressive degeneration of substantia nigra (SN) tyrosine hydroxylase (TH) expressing dopamine neurons (Qin et al. 2007). Another study comparing male and female C57BL/6J mice found that males show delayed (7 months) loss of SN dopaminergic neurons after a single LPS dose. However, females required multiple monthly LPS treatments that after 7 and 20 months later showed an approximate 40 to 50 % loss of SN TH-IR DA neurons and reduced rotor-rod ability that was transiently restored by l-dopa/carbidopa treatment (Liu et al. 2008). Neuroimmune signaling across and within brain cells likely contributes to loops of neuroimmune-NF-κB activation that include NADPH oxidase, the enzyme that makes reactive oxygen species described earlier (Qin and Crews 2012).

**Fig. 6:**
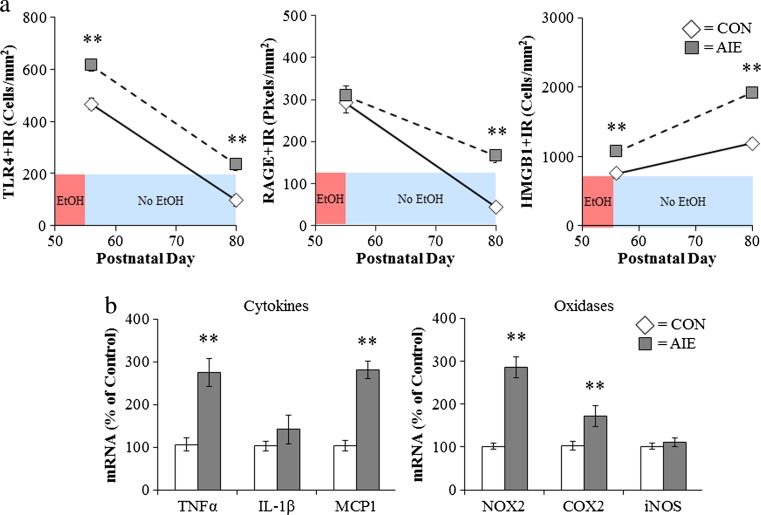
Adolescent intermittent ethanol (*AIE*) treatment leads to a persistent induction of neuroimmune genes in the adult brain. **a** Male Wistar rats were treated with ethanol (5 g/kg/day, i.g., *w*/*v*, 2 days on/2 days off) or comparable volumes of water from postnatal day (P)25 to P55. Brain tissue was collected either 24 h (P56) or 25 days after the last ethanol treatment (P80) to assess the persistent expression of neuroimmune markers. Toll-like receptor 4 (*TLR4*) immunoreactivity was upregulated 24 h after ethanol treatment and remained elevated for 25 days following the conclusion of ethanol treatment. In contrast, there was no change in receptor for advanced glycation end-product (*RAGE*) expression immediately following the conclusion of ethanol treatment but was elevated 25 days after the conclusion of ethanol treatment. Expression of high-mobility group box 1 (*HMGB1*), an endogenous TLR4 and RAGE agonist, was increased both 24 h and 25 days following the conclusion of ethanol treatment. **b** In a separate cohort of subjects, frontal cortex tissue samples were collected from CON- to AIE-treated animals on P80 (25 days following the conclusion of ethanol treatment) and neuroimmune gene mRNA levels were assessed. Adolescent binge ethanol treatment led to long-term upregulation of proinflammatory cytokines (tumor necrosis factor-alpha [*TNFα*] and monocyte chemotactic protein-1 [*MCP*-*1*]) and oxidases (cyclooxygenase [*COX-2*] and gp91^PHOX^ [*NOX2*]). **p < 0.01, relative to CONs. Data are adapted from Vetreno and Crews (2012) and Vetreno et al. (2013)

**Fig. 7:**
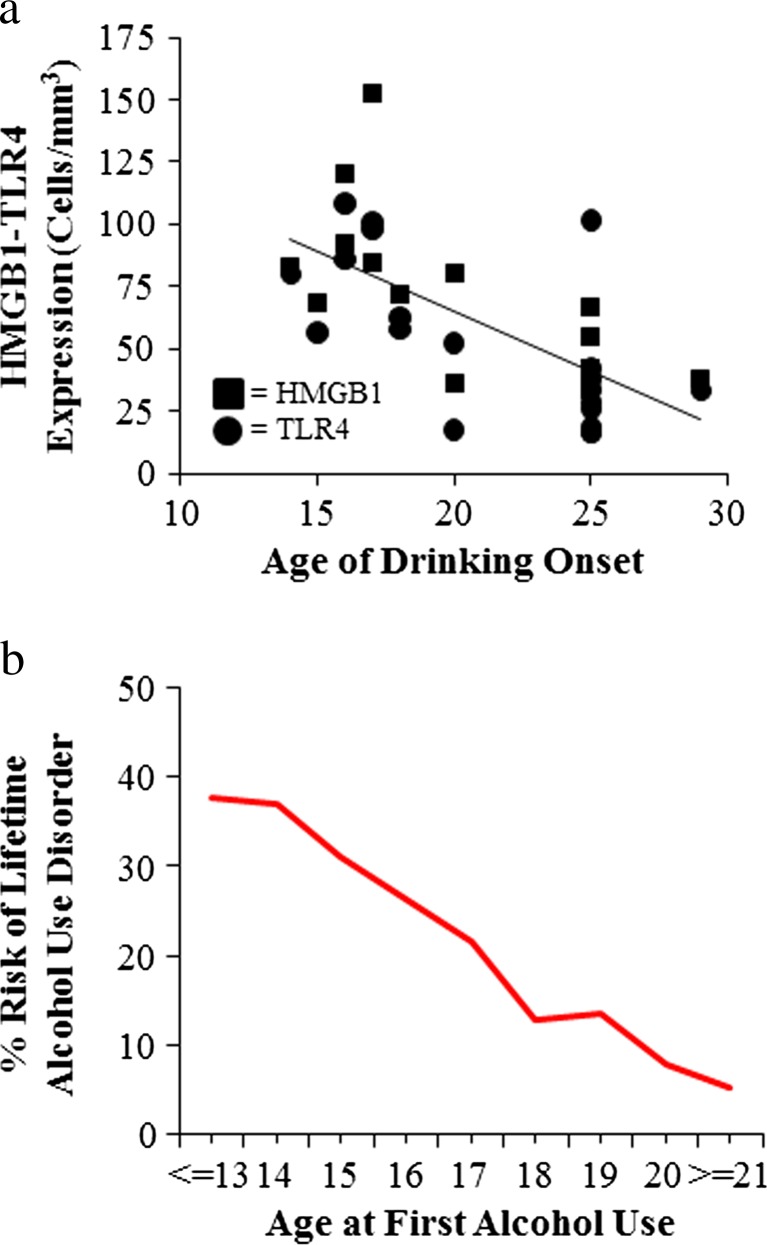
Risk of alcoholism and induction of innate immune genes correlate with age of drinking onset in humans. **a** Toll-like receptor 4 (*TLR4*) and high-mobility group box 1 (*HMGB1*) expression in the post-mortem human brain is negatively correlated with age of drinking onset adapted from Vetreno et al. (2013). **b** An earlier age of drinking onset is predictive of an increased likelihood of developing an alcohol use disorder during an individual’s lifetime. Adapted from Grant (1998)

Initial studies of alcohol and innate immune responses were confounded by the ethanol blockade of LPS-TLR4 signaling in monocytes and possibly other immune cells (Crews et al. 2006, 2011; Szabo and Mandrekar 2009). Szabo’s group reported that ethanol (25 mM) in vitro blunts LPS induction of TNFα (Szabo et al. 1995). Similarly, acute ethanol exposure attenuates TNFα, IL-1β, and IL-6 immune responses to LPS during intoxication (Pruett et al. 2004) that contrast with findings of in vivo chronic ethanol exposure modeling binge drinking that involve cycles of ethanol exposure and abstinence that cause progressive induction of HMGB1-TLR-RAGE expression and sensitization to neuroimmune activation. Indeed, models of moderate drinking find that multiple cycles cause transient neuroimmune induction in the brain (Whitman et al. 2013), whereas binge drinking models that utilize doses consistent with binge levels of ethanol (e.g., 5.0 g/kg, i.g.) induce persistent increases in the brain neuroimmune genes and proteins including HMGB1, TLR, and RAGE in adolescent rats (Vetreno and Crews 2012; Vetreno et al. 2013) and adult mice (Qin and Crews 2014; Qin et al. 2007, 2008). Similarly, studies of post-mortem human brain find that expression levels of HMGB1 and TLR in orbital frontal cortex (OFC) correlate with lifetime alcohol consumption across normal and alcoholic humans (Crews et al. 2013). This interesting correlation could only occur if ethanol induction of HMGB1-TLR receptors was persistent and cumulative with binge drinking episodes. Interestingly, treatment of adolescent rats modeling underage binge drinking induces HMGB1, TLR4, and RAGE that continue to undergo developmental changes in the brain (Fig. 6). HMGB1, TLR, and RAGE induction persists into abstinence, with TLR4 and RAGE showing developmental decreases in the frontal cortex and HMGB1 showing a developmental increase. Multiple other neuroimmune genes remain induced in adulthood (Fig. 6). Risk of alcohol dependence increases with a younger age on drinking onset (Fig. 7), and post-mortem prefrontal cortex expression also correlates with age of drinking onset (Fig. 7) (Vetreno et al. 2013). Together, these studies suggest that the persistent increase in brain HMGB1-TLR4 and neuroimmune signaling contribute to the chronic relapsing nature of alcoholism and the slow progressive degeneration found in alcoholism (Crews and Nixon 2009).

While induction of the innate immune system through NF-κB signaling cascades has been implicated in alcohol-induced neurodegeneration, recent evidence suggests that a shift in neurotrophic/innate immune signaling might also be involved. Cyclic AMP-responsive element binding protein (CREB) and many of its target genes, including neuropeptide Y (NPY) and brain-derived neurotrophic factor (BDNF), promote neuronal survival and protect neurons from excitotoxicity and apoptosis (Lonze and Ginty 2002). Levels of CREB-DNA binding, phosphorylated CREB, and BDNF are decreased in the rat frontal cortex following a 24-h withdrawal from chronic ethanol exposure (Pandey et al. 1999, 2001). Further, cortical levels of NPY are reduced by ethanol treatment, which was accompanied by a reduction of phosphorylated CREB (Bison and Crews 2003). Interestingly, our laboratory found that ethanol dose-dependently reduces CREB-DNA binding while simultaneously increasing NF-κB-DNA binding in hippocampal-entorhinal cortex slice culture (Zou and Crews 2006). However, following a prolonged period of abstinence, CREB level has been shown to rebound (Bison and Crews 2003), which could contribute to the recovery of white matter volume and cognitive function associated with protracted abstinence (Pfefferbaum et al. 1995; Sullivan et al. 2000a, b).

Although this review highlights HMGB1-TLR4 signaling, there are multiple other proinflammatory genes that are increased, and we have found many in the post-mortem human alcoholic brain (see Table 1). Initial studies found increased markers of microglia and the proinflammatory cytokine MCP1 (CCL2) post-mortem alcoholic ventral tegmental area, amygdala, nucleus accumbens, and hippocampus (He and Crews 2008). However, populations of activated microglia in other brain regions remain to be determined in the human post-mortem alcoholic brain. In studies focused on the PFC, we found that post-mortem alcoholic brain has increased levels of HMGB1 as well as TLR2, TLR3, and TLR4 receptors (Crews et al. 2013) and RAGE (Vetreno et al. 2013). Similarly, NADPH oxidase is increased in alcoholic PFC, the brain region most insulted in alcoholics (Crews and Nixon 2009; Qin and Crews 2012). In other studies focused on hippocampal neurogenesis, we found increased IL-1β inflammasome markers in the hippocampus of post-mortem alcoholic brain (Zou and Crews 2012). These studies indicate that multiple neuroimmune genes are upregulated in the human alcoholic brain and likely contribute to neurodegeneration and the neurobiology of alcoholism. These findings further support the role of neuroimmune signaling in human alcoholism and alcoholic neurodegeneration.

**Table 1:** Neuroimmune markers are increased in post-mortem human alcoholic brain

Marker	Full name	Effect	Citation
RAGE	Receptor for advanced glycation end products	↑	Vetreno et al. (2013)
TLR2	Toll-like receptor 2	↑	Crews et al. (2013)
TLR3	Toll-like receptor 3	↑	Crews et al. (2013)
TLR4	Toll-like receptor 4	↑	Crews et al. (2013)
HMGB1	High-mobility group box 1	↑	Crews et al. (2013)
IL-1β	Interleukin-1β	↑	Zou and Crews (2012)
NALP1	Nacht, leucine-rich repeat and pyrin domain containing protein	↑	Zou and Crews (2012)
gp91^phox^	NADPH oxidase 2	↑	Qin and Crews (2012)
MCP-1 (CCL2)	Monocyte chemotactic protein 1 (chemokine [C-C motif] ligand 2)	↑	He and Crews (2008)
Iba-1	Ionized calcium-binding adapter molecule 1	↑	He and Crews (2008)
GluT_5_	Microglia marker	↑	He and Crews (2008)
MK	Medkine	↑	Flatscher-Bader et al. (2005)

## Neuroimmune signaling, hyperexcitability, and neuronal death

Excitotoxicity is associated with alcoholic neurodegeneration and HMGB1-TLR4 signaling. Chronic ethanol treatment of neurons leads to increased sensitivity to excitotoxicity (Chandler et al. 1994). Ethanol potentiates glutamate excitotoxicity in brain slice cultures due to blockade of glial transporters (Zou and Crews 2010). However, in neuronal primary cultures, ethanol blocks *N*-methyl-d-aspartate (NMDA) excitotoxicity consistent with many studies finding ethanol inhibition of NMDA receptors (Chandler et al. 1998). Similar to acute ethanol blocking TLR4 receptor activation when present, ethanol blocks NMDA receptors when present (Chandler et al. 1998). Although ethanol can block NMDA responses, glutamate excitotoxicity is increased by ethanol and TNFα in brain slice cultures due in part to glial loss of glutamate uptake (Zou and Crews 2005) and perhaps release by ethanol. Further, HMGB1-TLR4 signaling has been shown to activate kinase cascades that lead to phosphorylation of the NR2B subunit of NMDA receptors causing the migration of more NMDA receptors to the synapse that increase synaptic NMDA receptors, neuronal excitability, and excitotoxicity (Balosso et al. 2014; Maroso et al. 2010). Both HMGB1-TLR4 signaling (Balosso et al. 2014) and IL1β-IL1R signaling (Viviani et al. 2003) have been shown to increase NMDA receptor-mediated calcium flux, neuronal excitability, and excitotoxicity through activation of kinase cascades. IL1β-IL1R activation of the Src kinase has been found to increase NMDA calcium flux, excitability, and excitotoxicity. Many studies have found that tyrosine-kinase activation can increase excitability through increases in NR2B-NMDA receptor phosphorylation. Dorit Ron’s group has found that ethanol increases NMDA excitability in hippocampus through kinase activation that alters receptor trafficking leading to increased NR2B-NMDA receptors and increased excitability (Suvarna et al. 2005). Another mechanism of chronic ethanol-induced hyperexcitability is neuroimmune inhibition of glial glutamate transporters (Zou and Crews 2005). Ethanol releases HMGB1 creating hyperexcitability that disrupts synaptic plasticity and sensitizes to excitotoxicity. HMGB1 is massively released during brain damage activating persistent neuroimmune gene induction (Kim et al. 2006). Indeed, Maroso et al. (2010) found increased release of HMGB1 with hippocampal excitability that caused seizures leading to persistent increases in HMGB1 and excitability. Ethanol has a modest cumulative effect that, with repeated chronic exposure, increases excitability and excitotoxicity due to increased neuroimmune signaling (see Fig. 4). Thus, the global neurodegeneration with the most severe losses in frontal cortex found in alcoholism is secondary to the persistent and progressive neuroimmune activation that occurs during alcoholism, which is a chronic relapsing disorder.

## Summary

Alcoholism is associated with increased neuroimmune gene expression in the brain. Neuroimmune gene induction appears to occur through two processes, systemic induction of innate immune genes resulting from alcohol-induced increases in gut permeability that result in increased blood cytokines that activate brain neuroimmune genes through multiple mechanisms including transport from blood into the brain. These signals activate neurons and glia through complex signaling that includes amplification through convergence of signaling through NF-κB and AP-1 pathways leading to the induction of proinflammatory cytokines, TLR receptors, RAGE, NADPH oxidase, and other oxidases. Ethanol also induces HMGB1 that contributes to positive loops of amplification of neuroimmune genes through TLR receptors and RAGE. Persistent activation of these pathways leads to a hyperexcitable state that disrupts neuronal networks contributing to alcoholic psychopathology as well as neurodegeneration. Agents that block ethanol neuroimmune activation may be able to prevent alcoholism and alcoholic neurodegeneration.
